# Prediction of functional outcome after mechanical thrombectomy using radiomics and deep learning features derived from intra-thrombus and peri-thrombus regions

**DOI:** 10.3389/fneur.2026.1943953

**Published:** 2026-09-14

**Authors:** Kai Shang, Xiaoxiao Xu, Lifang Ye, Yue Cheng, Jie Xiong, Yuehua Li, Jingxuan Jiang

**Affiliations:** 1 Institute of Diagnostic and Interventional Radiology, Shanghai Sixth People's Hospital Affiliated to Shanghai Jiao Tong University School of Medicine, Shanghai, China; 2 Department of Radiology, Wuxi No 2 People's Hospital, Wuxi, China; 3 Department of Radiology, The Tenth People's Hospital Affiliated to Tongji University, Shanghai, China; 4 Department of Cardiology, The First Affiliated Hospital, Jiangxi Medical College, Nanchang University, Nanchang, Jiangxi, China; 5 Department of Radiology, Affiliated Hospital of Nantong University, Nantong, China

**Keywords:** acute ischemic stroke, deep learning, mechanical thrombectomy, prognosis, radiomics, thrombus

## Abstract

**Purpose:**

Although mechanical thrombectomy (MT) achieves high recanalization rates in acute ischemic stroke (AIS), functional outcomes remain highly inconsistent. We aimed to evaluate the effectiveness of radiomics and deep learning features (DLFs) extracted from both intra-thrombus and peri-thrombus regions on baseline CT in predicting prognosis following MT.

**Materials and methods:**

This retrospective, multicenter study included 471 AIS patients who underwent MT. Patients from Center A (*n* = 315) constituted the training cohort, while those from Center B and C (*n* = 156) served as an external test cohort. Thrombus regions were manually segmented, with a subsequent 1 mm expansion to define the peri-thrombus regions. A total of 428 radiomics features and 128 DLFs were extracted from non-contrast CT (NCCT) and CTA images. After feature selection, ten machine learning classifiers were employed to build prediction models. Combined models were developed based on two strategies including early feature-level fusion and late decision-level fusion. Model performance was evaluated using ROC analysis, DeLong test, NRI and IDI. Subgroup analyses were also performed.

**Results:**

A total of 191 patients (40.6%) achieved a favorable outcome (90-day mRS ≤ 2). In the test cohort, the late-fusion combined model achieved the highest discriminative performance (AUC = 0.836, 95% CI: 0.769–0.891). It significantly outperformed both the peri-thrombus model (AUC = 0.758, *p* = 0.005) and the early-fusion combined model (AUC = 0.763, *p* = 0.009). Compared with the intra-thrombus model, the late-fusion combined model also demonstrated improved reclassification, with an NRI of 0.935 and an IDI of 0.278. Subgroup analyses showed consistent discriminative performance of the late-fusion combined model across the prespecified subgroups.

**Conclusion:**

The late-fusion combined model, integrating CT-derived radiomic and DLFs from both intra-thrombus and peri-thrombus regions, may provide an effective approach for predicting 90-day functional outcome after mechanical thrombectomy.

## Introduction

Stroke remains a leading cause of mortality and long-term disability worldwide, with ischemic stroke accounting for approximately 87% of all cases (1). Acute ischemic stroke (AIS) due to large vessel occlusion (LVO) represents a particularly severe subtype, comprising approximately 10 ~ 46% of AIS cases and conferring more than a twofold increased risk of mortality or long-term disability compared with non-LVO strokes (2). Mechanical thrombectomy (MT) has been recommended as the first-line treatment for LVO-AIS, with improved recanalization rates and functional outcomes (3). However, functional outcomes after MT remain highly heterogeneous, with nearly 50% of patients failing to achieve a favorable outcome despite successful recanalization, a phenomenon commonly termed futile recanalization (3, 4). In addition, some patients may develop malignant brain edema, a life-threatening complication associated with increased intracranial pressure, cerebral herniation, and high mortality (5). Therefore, accurate early prediction of treatment response and 90-day functional outcome is essential for risk stratification and individualized patient management.

Clinical outcomes in patients with AIS are influenced by a complex array of biological factors, including age, sex, and comorbidities, and clinical factors, such as stroke severity, blood pressure, blood glucose level, and time from stroke onset (6). However, comprehensive collection of clinical information can be challenging in the stroke emergency department workflow, whereas imaging biomarkers are more rapidly and reliably obtained. Several imaging-based markers, including Alberta Stroke Program Early CT Score (ASPECTS), collateral circulation scores, and post-procedural parenchymal hyperdensity, have been associated with functional outcomes after mechanical thrombectomy (7–9). As the understanding of thrombus characteristics has advanced, several thrombus-related imaging markers, including the hyperdense middle cerebral artery sign, clot burden score, and thrombus perviousness, have been reported to be associated with treatment response and clinical outcomes in patients with AIS undergoing MT (10–13). Although these markers provide valuable clinical information, their prognostic utility may be limited by suboptimal predictive performance and reliance on the subjective interpretation by radiologists. Therefore, developing a more precise and objective prediction model is necessary.

Radiomics enables high-throughput extraction of quantitative features from medical images, thereby capturing tissue characteristics beyond those discernible through visual assessment. When integrated with machine learning methods, these features can be used to develop predictive models that may improve risk stratification and support disease diagnosis, prognostication, and prediction of treatment response (14, 15). In stroke, CT-derived thrombus radiomics has shown potential in analyzing clot composition, estimating stroke onset time, and predicting outcomes (16–18). Nevertheless, radiomics features can be influenced by variations in image acquisition and processing (19). Deep learning (DL), particularly through convolutional neural networks (CNNs), can automatically learn hierarchical representations from medical images and identify complex patterns beyond handcrafted radiomic descriptors. It has demonstrated strong performance in image classification, detection, and segmentation tasks and may offer improvements in efficiency and reproducibility compared with conventional radiomics approaches (20, 21). However, deep learning features (DLFs) are often highly abstract and less interpretable than radiomics features. Integrating DLFs with radiomics features may therefore provide complementary information and improve model accuracy and reliability.

Previous studies have predominantly focused on intra-thrombus features, whereas the prognostic value of peri-thrombus characteristics remains insufficiently characterized. Therefore, this study aimed to evaluate the value of radiomics and DLFs derived from both intra-thrombus and peri-thrombus regions on pretreatment CT images for predicting 90-day functional outcomes in patients with LVO-AIS. We further compared multiple modeling strategies to identify an optimal approach for robust prognostic assessment.

## Materials and methods

### Study population

This retrospective study enrolled acute stroke patients from three medical centers between January 2018 and May 2025. The inclusion criteria included: (1) admission for persistent neurological deficits; (2) confirming anterior circulation LVO (terminal internal carotid artery or middle cerebral artery); (3) thrombus-related signs visible on admission NCCT or CTA; (4) subsequent immediate MT, with or without prior intravenous thrombolysis; (5) availability of complete clinical data and 90-day follow-up. The exclusion criteria included: (1) prior thrombectomy history; (2) inadequate image quality or insufficient thrombus size for analysis; (3) loss to follow-up. Demographics and clinical characteristics (including hypertension, diabetes, atrial fibrillation, coronary disease, admission NIHSS score, and occlusion site) were collected. The degree of recanalization after MT was assessed using the modified thrombolysis in cerebral infarction (mTICI). Successful recanalization was defined as mTICI ≥ 2b. Functional outcome was assessed using the modified Rankin Scale (mRS) at 90 days, with mRS ≤ 2 defined as a favorable outcome. The 90-day mRS was assessed via telephone interview by trained assessors who were blinded to the imaging findings and model predictions. Participants from Center A (*n* = 315) constituted the training cohort, and those from Center B and C (*n* = 156) formed the external test cohort. Figure 1 illustrated the participants selection flowchart.

**Figure 1:**
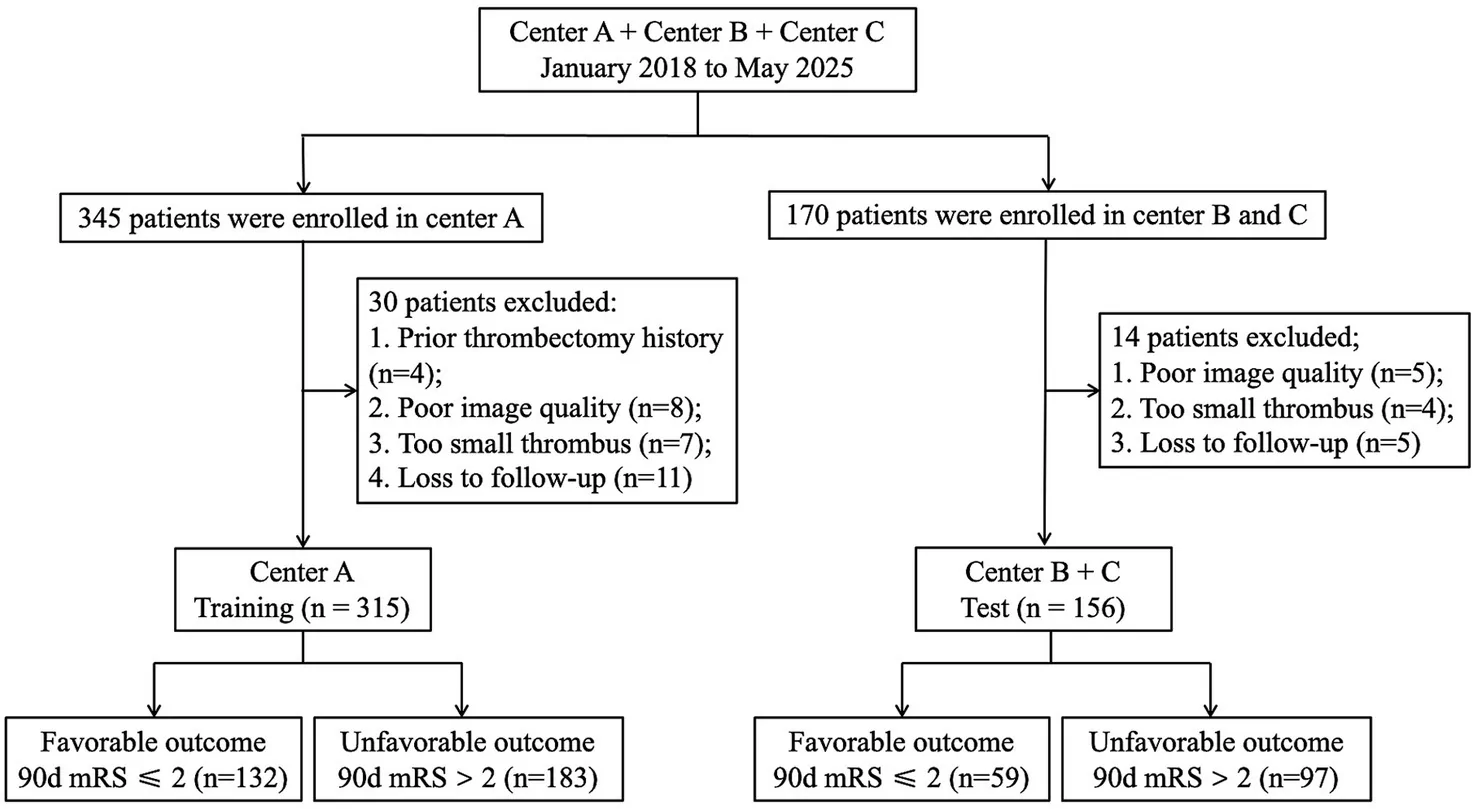
Flow chart of the participant selection process.

### CT data acquisition and thrombus segmentation

NCCT and CTA images were acquired using scanners from United Imaging (uCT960+), Philips (Brilliance iCT), and Siemens (SOMATOM Force), with reconstructed slice thicknesses between 0.63 and 1.00 mm. A radiologist manually delineated the thrombus slice by slice on CTA source images using ITK-SNAP software (version 5.4.0) to generate the intra-thrombus regions of interest (ROIs). Based on these initial ROIs, peri-thrombus ROIs were automatically generated by applying a 1-mm outward expansion to the original contours using Python (version 3.12.11). The CTA-derived intra-thrombus and peri-thrombus ROIs were subsequently transferred to the corresponding NCCT images through image registration, thereby defining the respective ROIs on NCCT. To assess segmentation reproducibility, 30 randomly selected cases were delineated twice by the same radiologist with a two-week interval and then independently delineated by a second radiologist.

### Radiomics and deep learning feature extraction

All images were normalized by scaling intensity values to a 0–600 HU range and resampling to a uniform resolution of 1 × 1 × 1 mm, to reduce heterogeneity arising from data acquired at different centers and with different CT scanners. Radiomics feature extraction was performed using the PyRadiomics toolkit.^1^ A total of 428 radiomics features were obtained from the intra-thrombus and peri-thrombus regions on both NCCT and CTA scans. The entire feature extraction procedure was performed in accordance with the Checklist for Evaluation of Radiomics research (CLEAR) guidelines.

To address the limitations of traditional radiomics features, we also extracted DLFs for fusion with radiomics. DLFs were derived using VGG-16 network. VGG-16 consists of 13 convolutional layers and 3 fully connected layers, with small 3 × 3 filters and a max pooling layer. Before DLF extraction, three consecutive axial slices centered on the cross-section with the largest thrombus area were selected and combined via channel-wise concatenation to form three-channel input data. All images were resized to 224 × 224 pixels to match the network input requirements. DLFs were extracted from the second fully connected layer (fc2) of the VGG-16. To mitigate the risk of overfitting due to the high dimensionality of the extracted features, dimensionality reduction was subsequently performed, resulting in a 32-dimensional DLF representation.

### Feature selection and classifier construction

DLFs and radiomics features were concatenated at the feature level and normalized using Z-score standardization to ensure uniform scaling. Feature selection involved four steps: (1) retaining features with an intraclass correlation coefficient (ICC) > 0.75; (2) selecting features with *p* < 0.05 by the independent samples t-test or Mann–Whitney U test; (3) for highly correlated feature pairs (Spearman correlation r > 0.90), the feature with a stronger correlation to the outcome was retained and the other was removed; (4) applying the least absolute shrinkage and selection operator (LASSO) regression with 5-fold cross-validation, where the optimal λ was determined by minimizing the mean squared error, retaining features with non-zero coefficients.

Ten supervised machine learning algorithms—Naive Bayes (NB), Support Vector Machine (SVM), K-Nearest Neighbors (KNN), Random Forest (RF), Extra Trees, Extreme Gradient Boosting (XGBoost), Light Gradient Boosting Machine (LightGBM), Gradient Boosting (GBoosting), Adaptive Boosting (AdaBoost), and Multi-layer Perceptron (MLP)—were utilized as classifiers. Two combined models integrating both intra-thrombus and peri-thrombus features were constructed using two strategies. For combined model 1, the DLFs and radiomics features from both regions on NCCT and CTA were concatenated into a single feature vector for subsequent analysis. For combined model 2, late fusion was implemented at the decision level using a stacking ensemble that integrated the best-performing intra-thrombus and peri-thrombus models. The workflow was presented in Figure 2.

**Figure 2:**
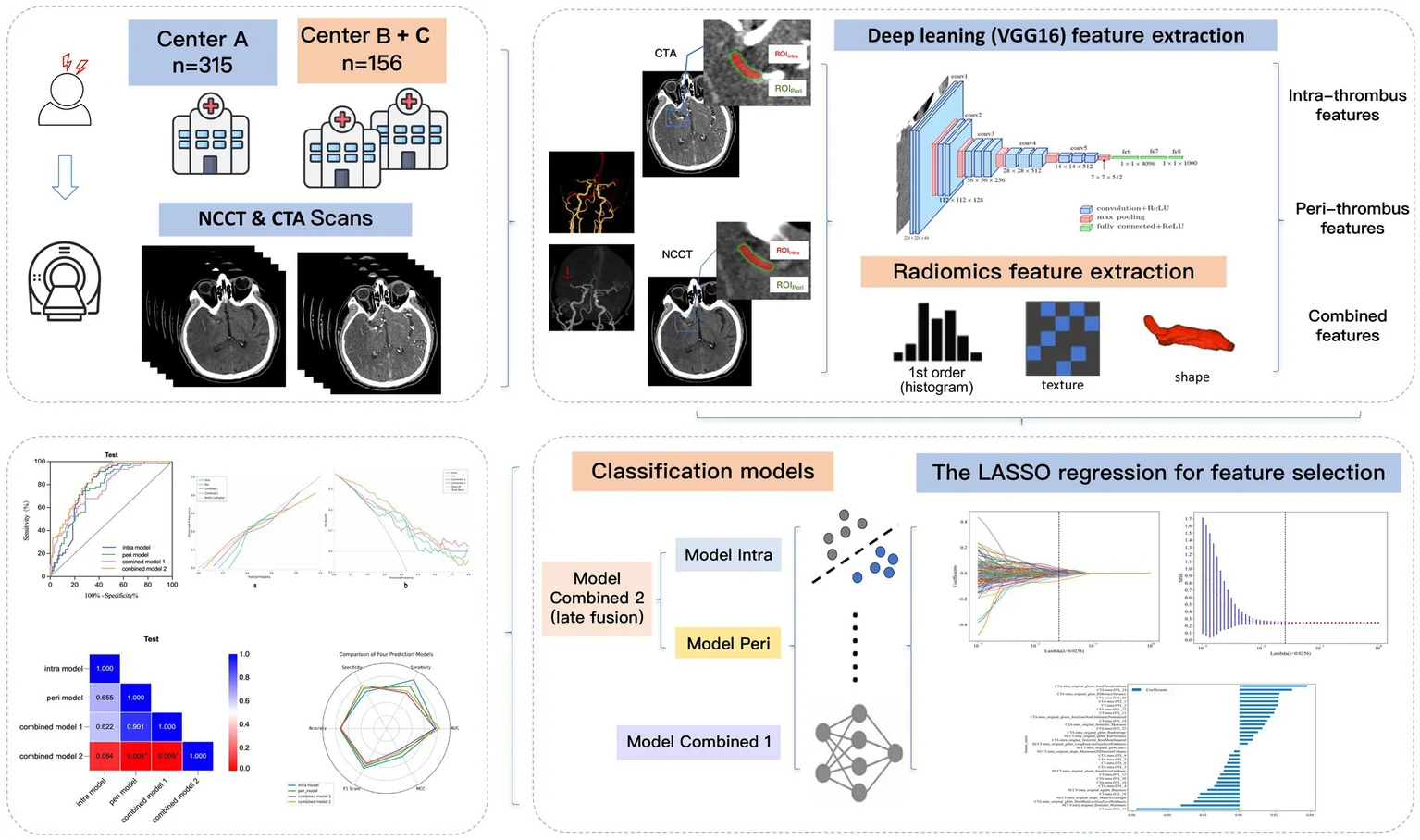
The workflow of this study.

### Model evaluation

Model performance was evaluated on both training and test cohorts using ROC curves. The optimal cutoff was determined by maximizing Youden’s index. Additional metrics included accuracy, sensitivity, specificity, positive predictive value (PPV), negative predictive value (NPV), and Matthews Correlation Coefficient (MCC). The DeLong’s test was employed to compare ROC curves. The shapley additive explanations (SHAP) analysis was performed to interpret the contribution of key features in the optimal models.

### Statistical analysis

Categorical variables were presented as frequencies (percentages). Continuous variables were expressed as mean ± standard deviation or median (interquartile range) based on normality (Shapiro–Wilk test). Group comparisons were performed using the Chi-square test or Fisher’s exact test for categorical variables and the T-test or Mann–Whitney U test for continuous variables. To analyze the correlations among features, Spearman correlation coefficient was employed. LASSO was used for feature selection. A two-sided *p* < 0.05 was considered statistically significant.

## Results

### Patient characteristics

The study finally enrolled 471 LVO-AIS patients. Patients were divided into a training cohort (center A, *n* = 315) and an external test cohort (center B and C, *n* = 156). 191 (40.6%) patients achieved favorable functional outcome (90-day mRS ≤ 2) following MT. Baseline clinical characteristics of the two cohorts were summarized in Table 1. Significant inter-cohort differences were observed in demographics and several risk factors. The test cohort had a higher proportion of men (62.18% vs. 50.48%, *p* = 0.02), a lower median age (70.00 [IQR: 64.00–75.50] vs. 74.00 [IQR: 64.00–82.00]; *p* < 0.01), and a lower median admission NIHSS score (12 [IQR: 6–15] vs. 14 [IQR: 9–19]; *p* < 0.01) compared with the training cohort. The test cohort also had a higher rate of smoking (25.64% vs. 13.65%, *p* < 0.01) but a lower rate of dyslipidemia (1.28% vs. 7.30%, *p* < 0.01). There were no significant differences between the two cohorts in degree of recanalization (mTICI) and favorable outcome.

**Table 1:** Baseline clinical characteristics of the participants.

Clinical characteristics	Training (*n* = 315)	Test (*n* = 156)	*p* value
Gender			0.02*
Male	159 (50.48)	97 (62.18)	
Female	156 (49.52)	59 (37.82)	
Age	74.00 (64.00, 82.00)	70.00 (64.00, 75.50)	<0.01*
Hypertension	203 (64.44)	97 (62.18)	0.63
Diabetes	82 (26.03)	42 (26.92)	0.84
Atrial fibrillation	118 (37.46)	60 (38.46)	0.85
Hyperlipidemia	23 (7.30)	2 (1.28)	<0.01*
Smoking	43 (13.65)	40 (25.64)	<0.01 *
NIHSS	14 (9, 19)	12 (6, 15)	<0.01*
Occlusion site			0.31
ICA	81 (25.71)	47 (30.13)	
MCA	234 (74.29)	109 (69.87)	
mTICI			0.06
mTICI ≥ 2b	265 (84.13)	120 (76.92)	
mTICI < 2b	50 (15.87)	36 (23.08)	
Clinical prognosis			0.40
90-day mRS ≤ 2	132 (41.90)	59 (37.82)	
90-day mRS > 2	183 (58.10)	97 (62.18)	

NIHSS, National Institutes of Health Stroke Scale; ICA, internal carotid artery; MCA, middle cerebral artery; mTICI, modified Treatment In Cerebral Ischemia; mRS, modified Rankin Scale. *p < 0.05.

### Feature extraction and selection

107 radiomics features and 32 DLFs were extracted for each ROI on NCCT and CTA. After concatenating deep learning and traditional radiomics features, a four-step feature selection process was applied. Finally, we identified 18 DLFs and 15 radiomics features for predicting favorable outcome in the intra-thrombus model, 13 DLFs and 14 radiomics features in the peri-thrombus model, and 20 DLFs and 16 radiomics features in the combined model 1. Feature selection was shown in Figure 3. Detailed information on the chosen DLFs and radiomic features could be found in the Supplementary Tables S1–S3.

**Figure 3:**
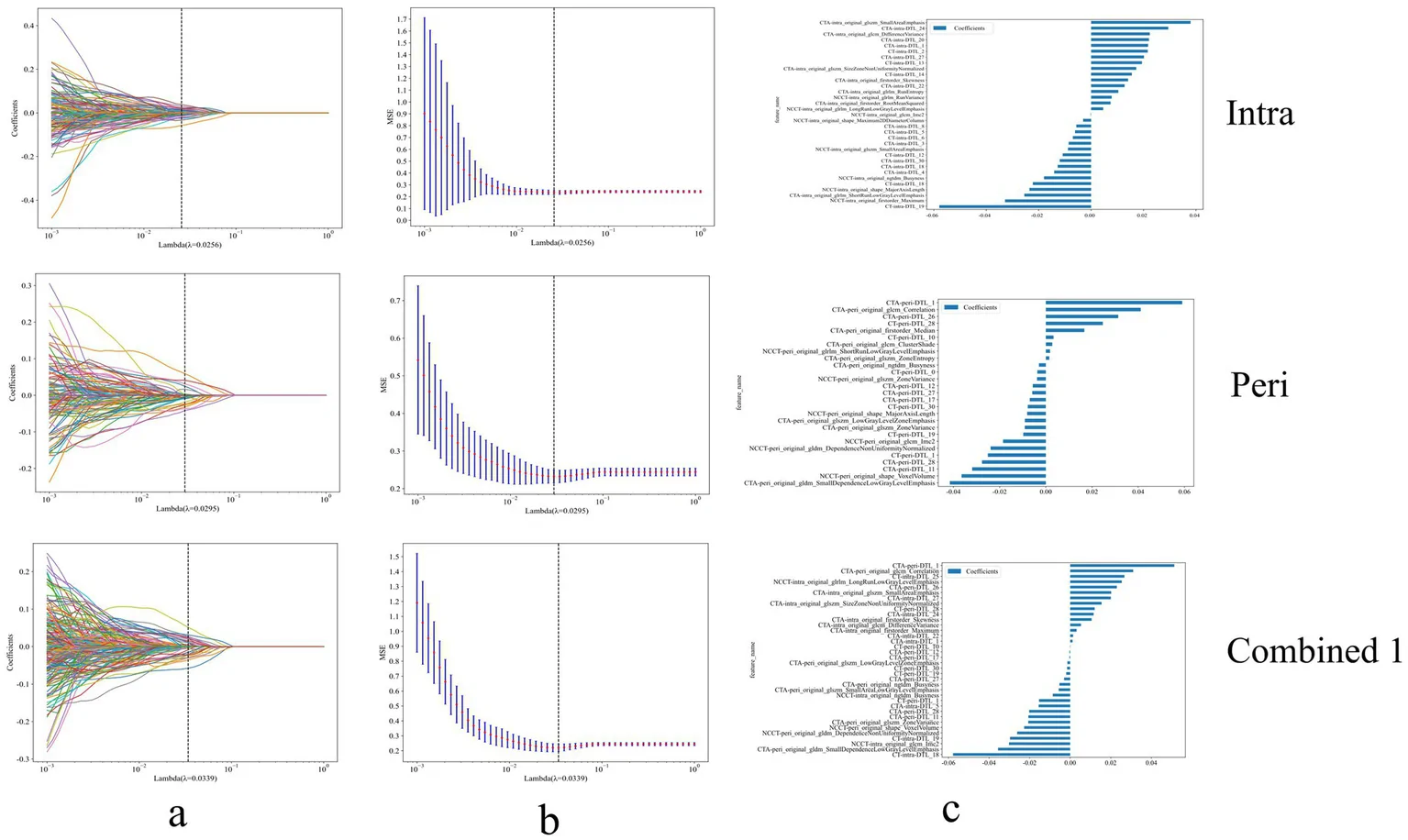
Feature selection in the intra-thrombus, peri-thrombus, and combined model 1 using the LASSO algorithm. **(a)** LASSO coefficient paths of the features. **(b)** Mean standard error (MSE) of 5-fold cross-validation. **(c)** Histogram of the score derived from the selected features.

### Performance and comparison of models

The performance of ten classifiers was evaluated in both the training and test cohorts, with detailed results presented in Supplementary Tables S4–S6 and Supplementary Figures S1–S3. In the training cohort, SVM-based models consistently achieved the best performance across all three configurations: the intra-thrombus model (AUC = 0.899, 95% CI = 0.862–0.935; Accuracy = 0.847; MCC = 0.685), the peri-thrombus model (AUC = 0.902, 95% CI = 0.868–0.937; Accuracy = 0.819; MCC = 0.660), and the combined model 1 (AUC = 0.946, 95% CI = 0.922–0.971; Accuracy = 0.879; MCC = 0.758).

In the test cohort, the SVM-based intra-thrombus model, peri-thrombus model, and combined model 1 continued to show the best performance. The intra-thrombus model achieved an AUC of 0.782 (95% CI: 0.711–0.854), accuracy of 0.731, and MCC of 0.500. The peri-thrombus model yielded an AUC of 0.758 (95% CI: 0.683–0.834), accuracy of 0.724, and MCC of 0.445. The combined model 1 attained an AUC of 0.763 (95% CI: 0.688–0.839), accuracy of 0.712, and MCC of 0.389. Figure 4 presented the SHAP plots for all three models.

**Figure 4:**
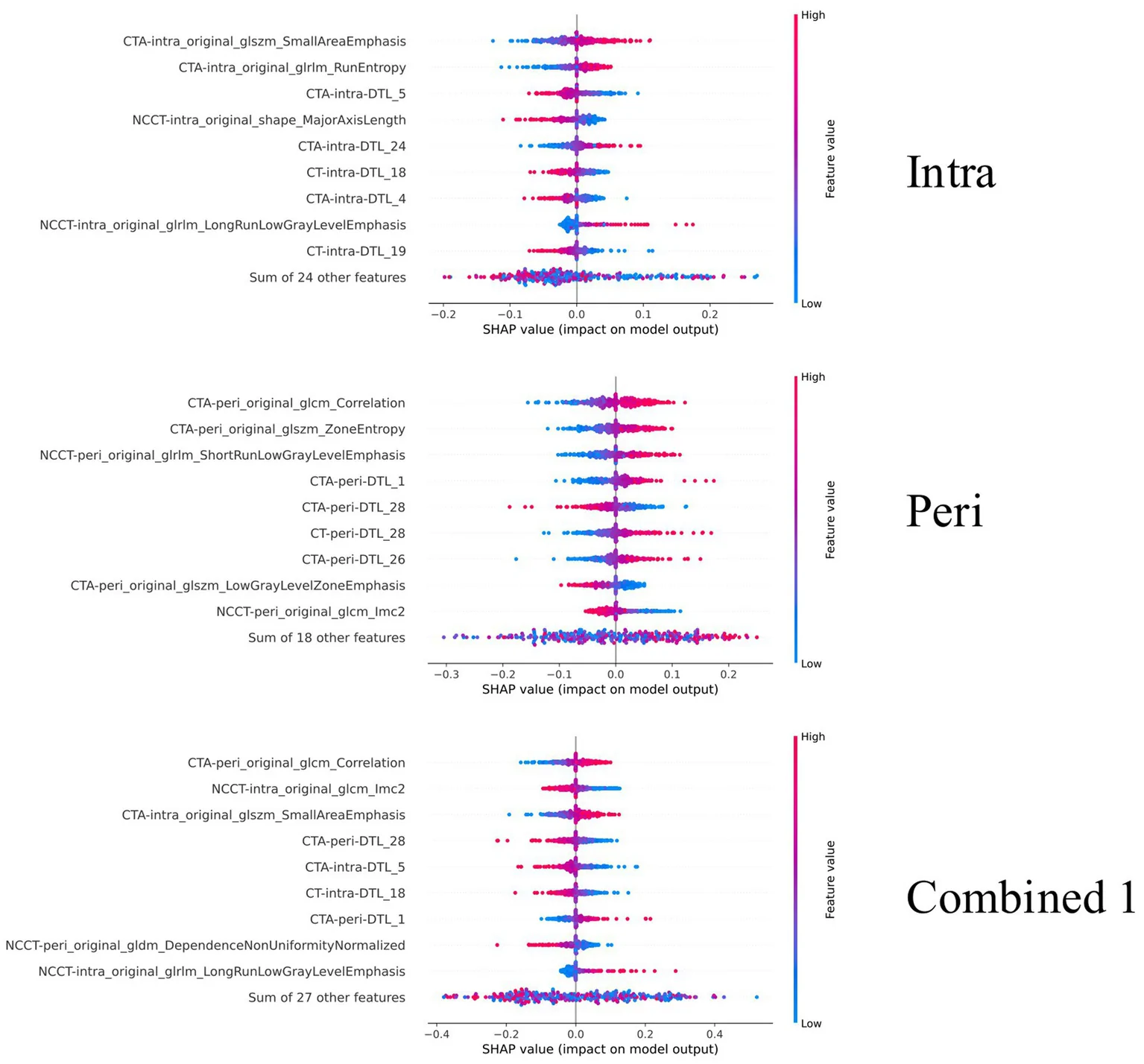
SHAP plots for the three models: Intra-thrombus, peri-thrombus, and combined model 1.

Combined Model 2 was constructed by decision-level integration of the best performing intra-thrombus and peri-thrombus models based on SVM. In the test cohort, it achieved an AUC of 0.836 (95% CI: 0.769–0.891), accuracy of 0.724, sensitivity of 0.661, specificity of 0.763, PPV of 0.629, NPV of 0.787, F1 score of 0.645, and MCC of 0.420.

Model comparison in the test cohort using the DeLong test showed that the combined model 2 significantly outperformed the peri-thrombus model (0.836 vs. 0.758, *p* = 0.005) and the combined model 1 (0.836 vs. 0.763, *p* = 0.009). Although its AUC was higher than that of the intra-thrombus model (0.836 vs. 0.782), the difference did not reach statistical significance (*p* = 0.084). However, compared with the intra-thrombus model, combined model 2 yielded positive NRI (0.935) and IDI (0.278). The model performance and DeLong test results were shown in Figure 5.

**Figure 5:**
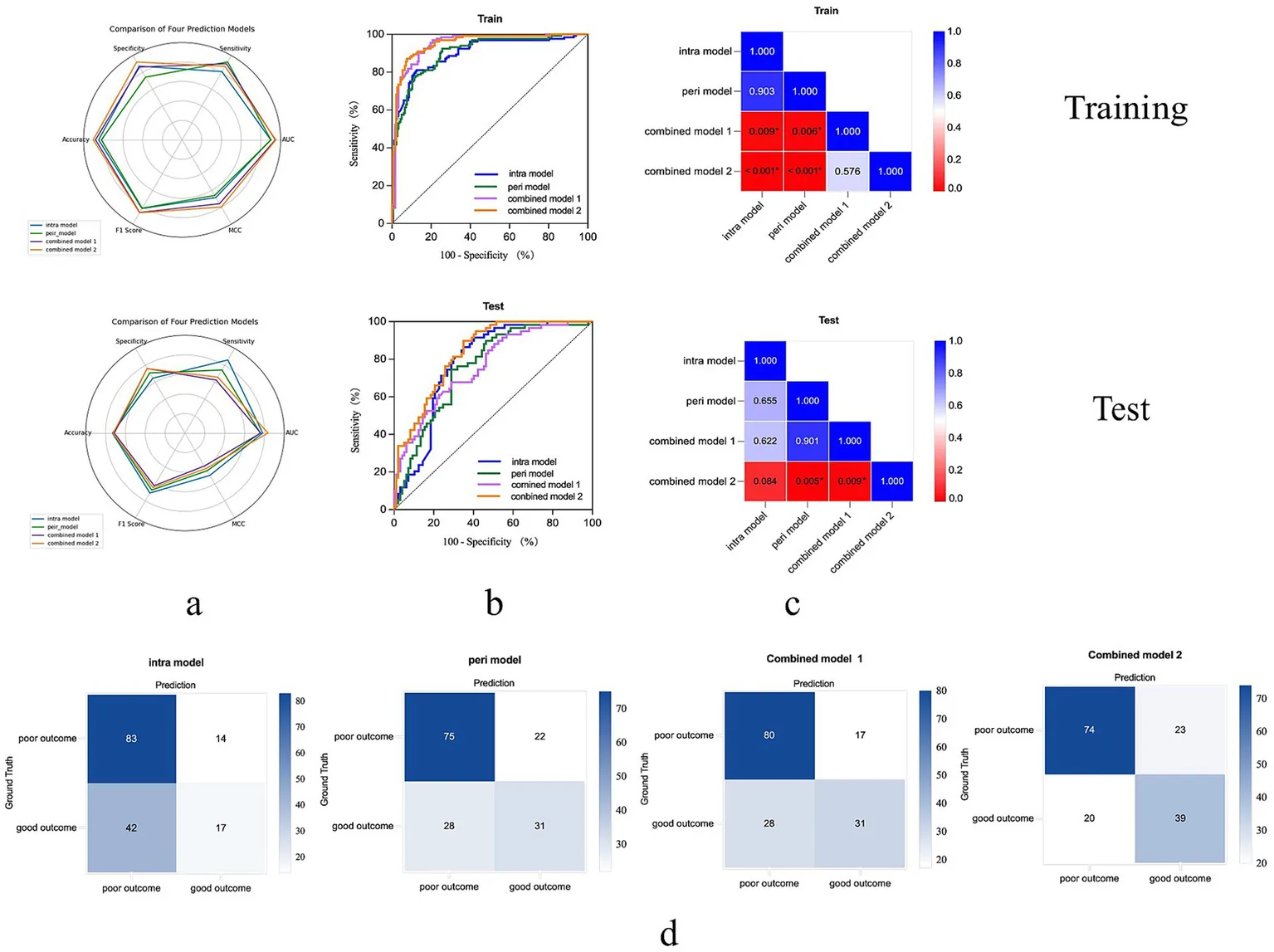
Comparisons of the performance results of the four models. **(a)** Performance of each model in the training and test cohorts. **(b)** ROC curves of each model in the training and test cohorts. **(c)** Results of the Delong test for each model in the training and test cohorts. **(d)** Confusion matrix of each model in the test cohort.

### Calibration and clinical utility of models

The calibration curve showed good agreement between prediction and observation for combined model 2 in test cohort. Decision curve analysis showed that combined model 2 offered the highest clinical net benefit across a broad range of threshold probabilities (Figure 6).

**Figure 6:**
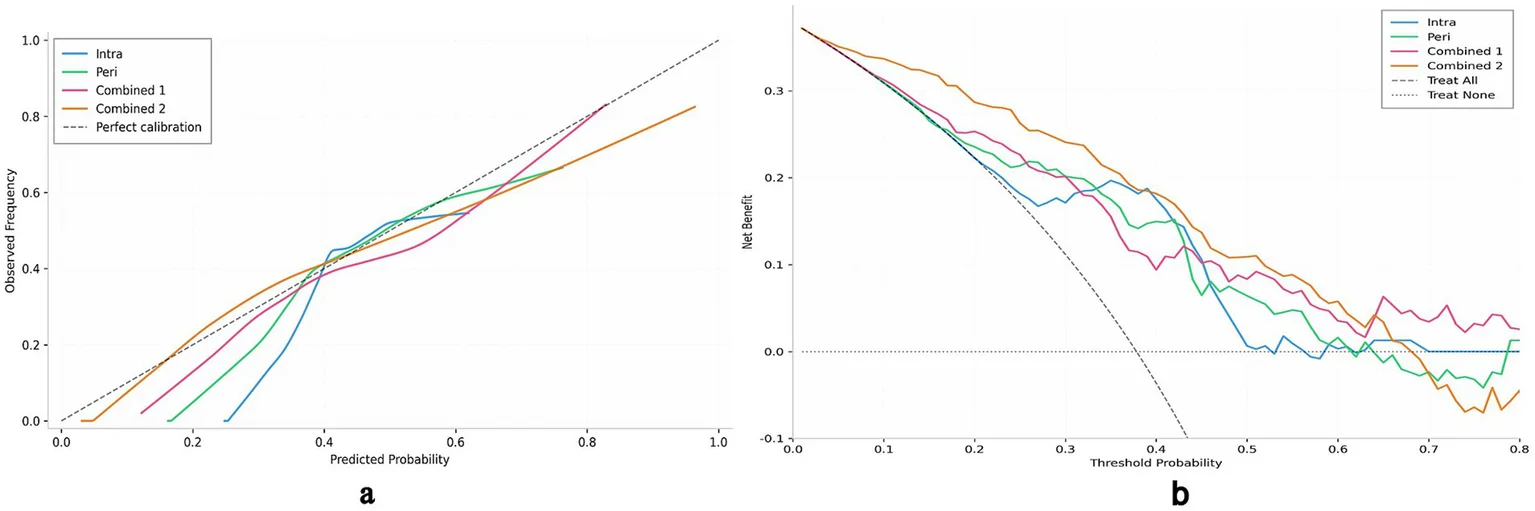
Calibration curves (**a**) and decision curve analysis (**b**) of the four models in the test cohort.

### Subgroup analysis

Subgroup analysis revealed that the combined model 2 generally maintained robust performance across subgroups defined by gender, age, and recanalization status, except in patients with NIHSS > 14, where the intra-thrombus model showed a higher AUC (Figure 7).

**Figure 7:**
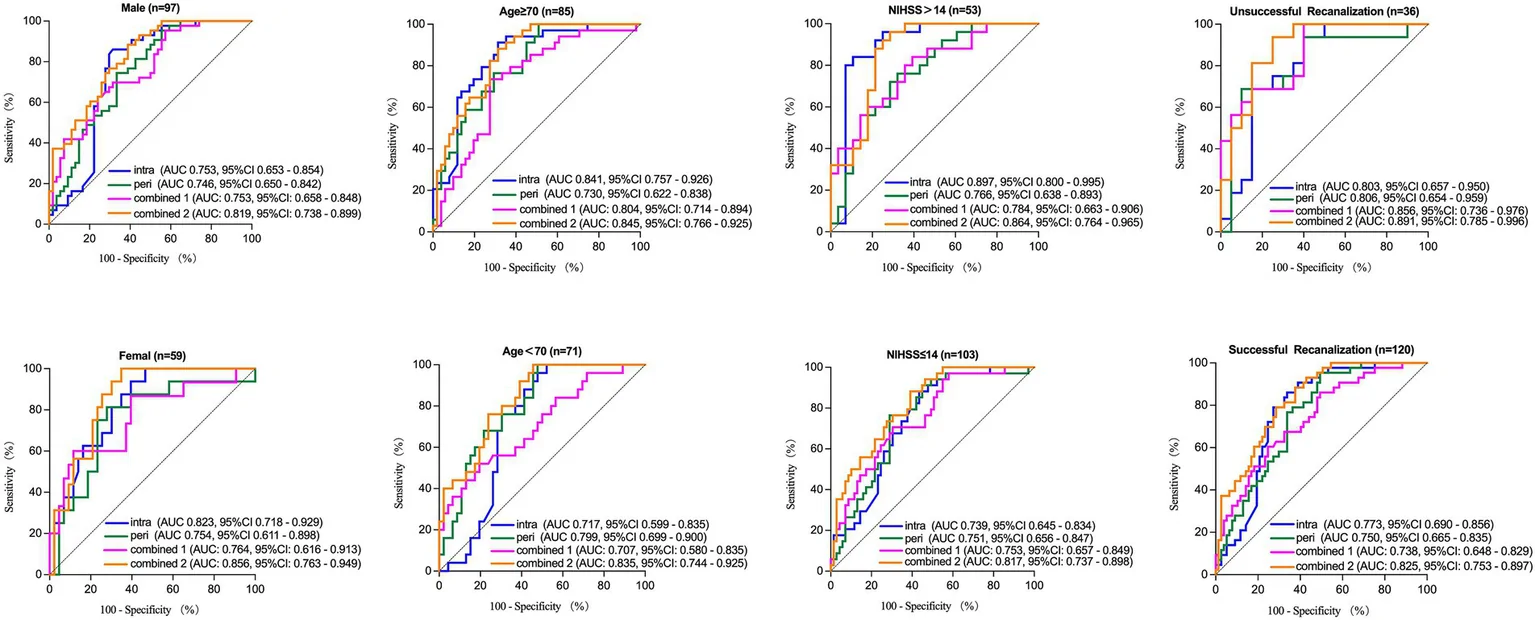
Subgroup analysis of model performance in test cohort.

## Discussion

Focusing on intra-thrombus and peri-thrombus regions, this study developed radiomics and deep learning models using multiple classifiers to predict functional outcomes after thrombectomy. Combined regional models were constructed via early-fusion and late-fusion strategies. The late-fusion combined model showed the best performance and remained stable across clinical subgroups, offering a potential non-invasive tool for individualized postoperative prognosis in stroke patients.

As the direct target of MT, the histological composition and physical properties of the thrombus significantly influence both the surgical procedure and the patient’s prognosis. Previous studies have shown that the histological composition of the thrombus was correlated with procedural parameters such as puncture-to-recanalization time, first-pass success rate, number of retrieval attempts, and the risk of distal embolization, which may further affect the patient’s functional outcome (22–24). Specifically, erythrocyte‑rich thrombi were more likely to achieve first-pass recanalization. This may be attributed to their lower stiffness and higher deformability, which may facilitate guidewire passage through the thrombus and reduce friction between the thrombus and vessel wall, thereby improving the efficiency of the thrombectomy procedure (25, 26). In contrast, fibrin/platelet-rich thrombi were firmer in texture and exhibited a higher coefficient of static friction with the vessel wall, increasing the difficulty of thrombectomy. Moreover, repeated and prolonged retrieval attempts could easily cause fragmentation of such thrombi, potentially leading to distal embolization (27–29). Therefore, imaging markers that enabled non-invasive assessment of thrombus composition and origin may provide important information for predicting distal embolization and prognosis after MT. However, traditional imaging markers were typically limited to indirect assessments of thrombus density or macroscopic morphology, and their measurements were highly dependent on the reader’s subjective judgment.

Deep learning and radiomics techniques provide a feasible approach for extracting high-dimensional features of the thrombus. This study integrated conventional radiomics features and DLFs, along with an analysis of peri-thrombus regions. The results showed that the intra-thrombus model exhibited stable predictive performance, further validating the significant impact of thrombus structural complexity on prognosis after MT. Moreover, the late fusion of the intra-thrombus and peri-thrombus models further enhanced the predictive capability, which showed the predictive value of the peri-thrombus regions for prognosis. We hypothesized that features derived from the peri-thrombus region may partially reflect the thrombus-vessel wall interface and pathological alterations in the adjacent vessel wall, such as atherosclerotic plaque formation, endothelial injury, and inflammatory infiltration. Previous studies have showed that intracranial atherosclerotic stenosis (ICAS) was responsible for approximately 34% of LVO-AIS. These ICAS-LVO cases were associated with higher rates of intraprocedural re-occlusion, longer puncture-to-recanalization time, and a slightly higher proportion of poor outcomes (30, 31). Thrombi related to ICAS usually attached to unstable plaque surfaces along with structural vessel wall alterations (32), and peri-thrombus features may capture this pattern.

This study evaluated and compared 10 different machine learning classifiers. The results showed that the SVM model performed best in both the training and test cohorts. The potential reasons were as follows: (1) this study involved high-dimensional data, and SVM excelled at finding the optimal separating hyperplane in high-dimensional spaces; (2) the sample size was relatively small, and SVM showed good generalization and robustness in small-sample scenarios; (3) SVM could effectively handle nonlinear relationships by mapping low-dimensional nonlinear data into a high-dimensional linearly separable space via kernel functions (33, 34).

This study compared the performance of models built with two fusion strategies and found that the late-fusion model outperformed the early fusion model, consistent with previous research (35), indicating that decision-level fusion was more robust in high-dimensional, small-sample settings. Late fusion allowed each regional model to learn region-specific patterns independently, thereby reducing noise propagation and overfitting, and improving generalizability. Subgroup analysis showed that, except for the subgroup with admission NIHSS score >14, the late-fusion model achieved higher AUC values across all other clinical subgroups, indicating good stability. In the high NIHSS score subgroup, the intra-thrombus model outperformed the late-fusion combined model, suggesting that imaging-based prognostic modeling should fully account for the potential moderating effects of clinical factors. Future studies with larger cohorts are needed to develop combined imaging-clinical models. The early-fusion combined model did not demonstrate improved performance compared with the individual region-based models. This finding may be related to differences in the distributions, scales, and noise characteristics of features extracted from the intra-thrombus and peri-thrombus regions. Direct concatenation of these features may introduce redundancy and multicollinearity, potentially diluting discriminative information and limiting the performance of the combined model.

This study had several limitations. First, the retrospective design may limit the applicability of the results. Further prospective validation in a larger cohort is needed. Second, the thrombus segmentation process was performed manually, which is time-consuming and may affect the reliability of the results. Furthermore, the peri-thrombus region was defined by a fixed 1 mm expansion; other definitions might encompass different biological processes. Future studies should focus on developing automated or semi-automated methods for more efficient and accurate thrombus segmentation to confirm these findings and explore optimal peri-thrombus definitions. Third, although image normalization and cross-validation were performed, potential bias may exist as our training and test cohorts were from different centers.

## Conclusion

A predictive model integrating deep learning and radiomics features derived from intra-thrombus and peri-thrombus regions on admission CT, particularly when using a late-fusion strategy, demonstrated favorable performance in predicting functional outcomes after MT for LVO-AIS. This approach may provide a noninvasive tool for individualized risk stratification and may assist clinical decision-making.

## Data Availability

The original contributions presented in the study are included in the article/Supplementary material, further inquiries can be directed to the corresponding authors.

## Footnotes

1
https://pypi.org/project/pyradiomic/
