# Supplementary material for: Prediction of functional outcome after mechanical thrombectomy using radiomics and deep learning features derived from intra-thrombus and peri-thrombus regions
Source: Front Neurol. 2026 Sep 14;17:1943953. doi: 10.3389/fneur.2026.1943953 (PMC13616720; doi:10.3389/fneur.2026.1943953)
Supplement: Supplementary file 1 [file Supplementary_file_1.DOCX]

Table S1 Intra-thrombus deep learning and radiomics features

| Scan Type | Feature Category | Feature Name |
| --- | --- | --- |
| CTA | Deep Learning Features | CTA-intra-DTL_1 |
|  |  | CTA-intra-DTL_3 |
|  |  | CTA-intra-DTL_4 |
|  |  | CTA-intra-DTL_5 |
|  |  | CTA-intra-DTL_8 |
|  |  | CTA-intra-DTL_18 |
|  |  | CTA-intra-DTL_20 |
|  |  | CTA-intra-DTL_22 |
|  |  | CTA-intra-DTL_24 |
|  |  | CTA-intra-DTL_27 |
|  |  | CTA-intra-DTL_30 |
|  | Radiomics Features | CTA-intra_original_firstorder_RootMeanSquared |
|  |  | CTA-intra_original_firstorder_Skewness |
|  |  | CTA-intra_original_glcm_DifferenceVariance |
|  |  | CTA-intra_original_glrlm_RunEntropy |
|  |  | CTA-intra_original_glrlm_ShortRunLowGrayLevelEmphasis |
|  |  | CTA-intra_original_glszm_SizeZoneNonUniformityNormalized |
|  |  | CTA-intra_original_glszm_SmallAreaEmphasis |
| NCCT | Deep Learning Features | CT-intra-DTL_2 |
|  |  | CT-intra-DTL_6 |
|  |  | CT-intra-DTL_12 |
|  |  | CT-intra-DTL_13 |
|  |  | CT-intra-DTL_14 |
|  |  | CT-intra-DTL_18 |
|  |  | CT-intra-DTL_19 |
|  | Radiomics Features | NCCT-intra_original_firstorder_Maximum |
|  |  | NCCT-intra_original_glcm_Imc2 |
|  |  | NCCT-intra_original_glrlm_LongRunLowGrayLevelEmphasis |
|  |  | NCCT-intra_original_glrlm_RunVariance |
|  |  | NCCT-intra_original_glszm_SmallAreaEmphasis |
|  |  | NCCT-intra_original_ngtdm_Busyness |
|  |  | NCCT-intra_original_shape_MajorAxisLength |
|  |  | NCCT-intra_original_shape_Maximum2DDiameterColumn |

Table S2 Peri-thrombus deep learning and radiomics features

| Scan Type | Feature Category | Feature Name |
| --- | --- | --- |
| CTA | Deep Learning Features | CTA-peri-DTL_1 |
|  |  | CTA-peri-DTL_11 |
|  |  | CTA-peri-DTL_12 |
|  |  | CTA-peri-DTL_17 |
|  |  | CTA-peri-DTL_26 |
|  |  | CTA-peri-DTL_27 |
|  |  | CTA-peri-DTL_28 |
|  | Radiomics Features | CTA-peri_original_firstorder_Median |
|  |  | CTA-peri_original_glcm_ClusterShade |
|  |  | CTA-peri_original_glcm_Correlation |
|  |  | CTA-peri_original_gldm_SmallDependenceLowGrayLevelEmphasis |
|  |  | CTA-peri_original_glszm_LowGrayLevelZoneEmphasis |
|  |  | CTA-peri_original_glszm_ZoneEntropy |
|  |  | CTA-peri_original_glszm_ZoneVariance |
|  |  | CTA-peri_original_ngtdm_Busyness |
| NCCT | Deep Learning Features | CT-peri-DTL_0 |
|  |  | CT-peri-DTL_1 |
|  |  | CT-peri-DTL_10 |
|  |  | CT-peri-DTL_19 |
|  |  | CT-peri-DTL_28 |
|  |  | CT-peri-DTL_30 |
|  | Radiomics Features | NCCT-peri_original_glrlm_ShortRunLowGrayLevelEmphasis |
|  |  | NCCT-peri_original_glszm_ZoneVariance |
|  |  | NCCT-peri_original_shape_MajorAxisLength |
|  |  | NCCT-peri_original_shape_VoxelVolume |

Table S3 Combined model 1 deep learning and radiomics features

| Scan Type | Feature Category |  | Feature Name |
| --- | --- | --- | --- |
| CTA | Deep Learning Features | intra | CTA-intra-DTL_1 |
|  |  |  | CTA-intra-DTL_5 |
|  |  |  | CTA-intra-DTL_22 |
|  |  |  | CTA-intra-DTL_24 |
|  |  |  | CTA-intra-DTL_27 |
|  |  | peri | CTA-peri-DTL_1 |
|  |  |  | CTA-peri-DTL_11 |
|  |  |  | CTA-peri-DTL_12 |
|  |  |  | CTA-peri-DTL_17 |
|  |  |  | CTA-peri-DTL_26 |
|  |  |  | CTA-peri-DTL_27 |
|  |  |  | CTA-peri-DTL_28 |
|  | Radiomics Features | intra | CTA-intra_original_firstorder_Maximum; |
|  |  |  | CTA-intra_original_firstorder_Skewness; |
|  |  |  | CTA-intra_original_glcm_DifferenceVariance; |
|  |  |  | CTA-intra_original_glszm_SizeZoneNonUniformityNormalized; |
|  |  |  | CTA-intra_original_glszm_SmallAreaEmphasis |
|  |  | peri | CTA-peri_original_glcm_Correlation; |
|  |  |  | CTA-peri_original_gldm_SmallDependenceLowGrayLevelEmphasis; |
|  |  |  | CTA-peri_original_glszm_LowGrayLevelZoneEmphasis; |
|  |  |  | CTA-peri_original_glszm_SmallAreaLowGrayLevelEmphasis; |
|  |  |  | CTA-peri_original_glszm_ZoneVariance; |
|  |  |  | CTA-peri_original_ngtdm_Busyness |
| NCCT | Deep Learning Features | intra | CT-intra-DTL_18 |
|  |  |  | CT-intra-DTL_19 |
|  |  |  | CT-intra-DTL_25 |
|  |  | peri | CT-peri-DTL_1 |
|  |  |  | CT-peri-DTL_10 |
|  |  |  | CT-peri-DTL_19 |
|  |  |  | CT-peri-DTL_28 |
|  |  |  | CT-peri-DTL_30 |
|  | Radiomics Features | intra | NCCT-intra_original_glcm_Imc2; |
|  |  |  | NCCT-intra_original_glrlm_LongRunLowGrayLevelEmphasis; |
|  |  |  | NCCT-intra_original_ngtdm_Busyness |
|  |  | peri | NCCT-peri_original_gldm_DependenceNonUniformityNormalized; |
|  |  |  | NCCT-peri_original_shape_VoxelVolume |

Table S4 ROC curve analysis of different classifiers for the intra-thrombus model

| Cohort | Classifier | Accuracy | AUC | 95% CI | Sensitivity | Specificity | PPV | NPV | F1-Score | MCC |
| --- | --- | --- | --- | --- | --- | --- | --- | --- | --- | --- |
| Training | RandomForest | 0.990 | 0.999 | 0.9965 - 1.0000 | 1.000 | 0.984 | 0.978 | 1.000 | 0.989 | 0.981 |
|  | ExtraTrees | 1.000 | 1.000 | 1.0000 - 1.0000 | 1.000 | 1.000 | 1.000 | 1.000 | 1.000 | 1.000 |
|  | KNN | 0.777 | 0.834 | 0.7916 - 0.8774 | 0.740 | 0.803 | 0.729 | 0.812 | 0.735 | 0.543 |
|  | SVM | 0.847 | 0.899 | 0.8621 - 0.9351 | 0.809 | 0.874 | 0.822 | 0.865 | 0.815 | 0.685 |
|  | NaiveBayes | 0.685 | 0.749 | 0.6951 - 0.8035 | 0.786 | 0.612 | 0.592 | 0.800 | 0.675 | 0.395 |
|  | GradientBoosting | 0.869 | 0.936 | 0.9106 - 0.9621 | 0.863 | 0.874 | 0.831 | 0.899 | 0.846 | 0.733 |
|  | LightGBM | 0.927 | 0.976 | 0.9626 - 0.9887 | 0.908 | 0.940 | 0.915 | 0.935 | 0.912 | 0.849 |
|  | MLP | 0.768 | 0.844 | 0.8024 - 0.8865 | 0.763 | 0.770 | 0.704 | 0.820 | 0.733 | 0.529 |
|  | XGBoost | 1.000 | 1.000 | 1.0000 - 1.0000 | 1.000 | 1.000 | 1.000 | 1.000 | 1.000 | 1.000 |
|  | AdaBoost | 0.726 | 0.837 | 0.7939 - 0.8796 | 0.863 | 0.628 | 0.624 | 0.865 | 0.724 | 0.490 |
| Test | RandomForest | 0.513 | 0.611 | 0.5239 - 0.6973 | 0.831 | 0.320 | 0.426 | 0.756 | 0.563 | 0.165 |
|  | ExtraTrees | 0.609 | 0.642 | 0.5548 - 0.7295 | 0.661 | 0.577 | 0.487 | 0.737 | 0.561 | 0.231 |
|  | KNN | 0.577 | 0.647 | 0.5620 - 0.7328 | 0.780 | 0.454 | 0.465 | 0.772 | 0.582 | 0.235 |
|  | SVM | 0.731 | 0.782 | 0.7107 - 0.8543 | 0.864 | 0.649 | 0.600 | 0.887 | 0.708 | 0.500 |
|  | NaiveBayes | 0.705 | 0.714 | 0.6282 - 0.7994 | 0.763 | 0.670 | 0.584 | 0.823 | 0.662 | 0.420 |
|  | GradientBoosting | 0.679 | 0.646 | 0.5579 - 0.7339 | 0.339 | 0.887 | 0.645 | 0.688 | 0.444 | 0.274 |
|  | LightGBM | 0.628 | 0.592 | 0.4993 - 0.6844 | 0.441 | 0.742 | 0.510 | 0.686 | 0.473 | 0.189 |
|  | MLP | 0.705 | 0.698 | 0.6144 - 0.7810 | 0.678 | 0.722 | 0.597 | 0.787 | 0.635 | 0.391 |
|  | XGBoost | 0.603 | 0.644 | 0.5555 - 0.7330 | 0.746 | 0.515 | 0.484 | 0.769 | 0.587 | 0.257 |
|  | AdaBoost | 0.609 | 0.592 | 0.4995 - 0.6840 | 0.559 | 0.639 | 0.485 | 0.705 | 0.520 | 0.194 |

AUC: area under the curve, 95% CI: 95% confidence intervals, PPV: positive predictive values, NPV: negative predictive values, MCC: Matthews Correlation Coefficient.

Table S5 ROC curve analysis of different classifiers for the peri-thrombus model

| Cohort | Classifier | Accuracy | AUC | 95% CI | Sensitivity | Specificity | PPV | NPV | F1-Score | MCC |
| --- | --- | --- | --- | --- | --- | --- | --- | --- | --- | --- |
| Training | RandomForest | 0.981 | 0.998 | 0.9955 - 1.0000 | 0.977 | 0.984 | 0.977 | 0.984 | 0.977 | 0.961 |
|  | ExtraTrees | 1.000 | 1.000 | 1.0000 - 1.0000 | 1.000 | 1.000 | 1.000 | 1.000 | 1.000 | 1.000 |
|  | KNN | 0.768 | 0.813 | 0.7682 - 0.8581 | 0.659 | 0.847 | 0.757 | 0.775 | 0.704 | 0.519 |
|  | SVM | 0.819 | 0.902 | 0.8681 - 0.9365 | 0.924 | 0.743 | 0.722 | 0.932 | 0.811 | 0.660 |
|  | NaiveBayes | 0.721 | 0.766 | 0.7131 - 0.8185 | 0.803 | 0.661 | 0.631 | 0.823 | 0.707 | 0.459 |
|  | GradientBoosting | 0.848 | 0.915 | 0.8850 - 0.9442 | 0.750 | 0.918 | 0.868 | 0.836 | 0.805 | 0.686 |
|  | LightGBM | 0.937 | 0.979 | 0.9660 - 0.9916 | 0.902 | 0.962 | 0.944 | 0.931 | 0.922 | 0.869 |
|  | MLP | 0.778 | 0.852 | 0.8111 - 0.8932 | 0.735 | 0.809 | 0.735 | 0.809 | 0.735 | 0.544 |
|  | XGBoost | 1.000 | 1.000 | 1.0000 - 1.0000 | 1.000 | 1.000 | 1.000 | 1.000 | 1.000 | 1.000 |
|  | AdaBoost | 0.752 | 0.817 | 0.7704 - 0.8626 | 0.780 | 0.732 | 0.678 | 0.822 | 0.725 | 0.506 |
| Test | RandomForest | 0.615 | 0.621 | 0.5293 - 0.7136 | 0.610 | 0.619 | 0.493 | 0.723 | 0.545 | 0.222 |
|  | ExtraTrees | 0.660 | 0.685 | 0.6014 - 0.7692 | 0.559 | 0.722 | 0.550 | 0.729 | 0.555 | 0.280 |
|  | KNN | 0.577 | 0.593 | 0.5029 - 0.6823 | 0.525 | 0.608 | 0.449 | 0.678 | 0.484 | 0.131 |
|  | SVM | 0.724 | 0.758 | 0.6831 - 0.8336 | 0.746 | 0.711 | 0.611 | 0.821 | 0.672 | 0.445 |
|  | NaiveBayes | 0.667 | 0.703 | 0.6192 - 0.7871 | 0.678 | 0.660 | 0.548 | 0.771 | 0.606 | 0.328 |
|  | GradientBoosting | 0.686 | 0.669 | 0.5802 - 0.7570 | 0.593 | 0.742 | 0.583 | 0.750 | 0.588 | 0.334 |
|  | LightGBM | 0.692 | 0.668 | 0.5779 - 0.7581 | 0.593 | 0.753 | 0.593 | 0.753 | 0.593 | 0.346 |
|  | MLP | 0.692 | 0.727 | 0.6448 - 0.8097 | 0.729 | 0.670 | 0.573 | 0.802 | 0.642 | 0.387 |
|  | XGBoost | 0.654 | 0.687 | 0.6003 - 0.7745 | 0.712 | 0.619 | 0.532 | 0.779 | 0.609 | 0.320 |
|  | AdaBoost | 0.615 | 0.598 | 0.5065 - 0.6885 | 0.678 | 0.577 | 0.494 | 0.747 | 0.571 | 0.248 |

AUC: area under the curve, 95% CI: 95% confidence intervals, PPV: positive predictive values, NPV: negative predictive values, MCC: Matthews Correlation Coefficient.

Table S6 ROC curve analysis of different classifiers for combined model 1

| Cohort | Classifier | Accuracy | AUC | 95% CI | Sensitivity | Specificity | PPV | NPV | F1-Score | MCC |
| --- | --- | --- | --- | --- | --- | --- | --- | --- | --- | --- |
| Training | RandomForest | 0.984 | 0.997 | 0.9922 - 1.0000 | 0.985 | 0.984 | 0.977 | 0.989 | 0.981 | 0.967 |
|  | ExtraTrees | 1.000 | 1.000 | 1.0000 - 1.0000 | 1.000 | 1.000 | 1.000 | 1.000 | 1.000 | 1.000 |
|  | KNN | 0.765 | 0.841 | 0.7991 - 0.8819 | 0.674 | 0.831 | 0.742 | 0.779 | 0.706 | 0.513 |
|  | SVM | 0.879 | 0.946 | 0.9215 - 0.9714 | 0.902 | 0.863 | 0.826 | 0.924 | 0.862 | 0.758 |
|  | NaiveBayes | 0.746 | 0.811 | 0.7628 - 0.8593 | 0.818 | 0.694 | 0.659 | 0.841 | 0.730 | 0.506 |
|  | GradientBoosting | 0.914 | 0.967 | 0.9489 - 0.9844 | 0.909 | 0.918 | 0.889 | 0.933 | 0.899 | 0.825 |
|  | LightGBM | 0.943 | 0.982 | 0.9693 - 0.9939 | 0.909 | 0.967 | 0.952 | 0.937 | 0.930 | 0.883 |
|  | MLP | 0.844 | 0.915 | 0.8852 - 0.9452 | 0.864 | 0.831 | 0.786 | 0.894 | 0.823 | 0.687 |
|  | XGBoost | 1.000 | 1.000 | 1.0000 - 1.0000 | 1.000 | 1.000 | 1.000 | 1.000 | 1.000 | 1.000 |
|  | AdaBoost | 0.746 | 0.849 | 0.8088 - 0.8902 | 0.909 | 0.628 | 0.638 | 0.906 | 0.750 | 0.541 |
| Test | RandomForest | 0.532 | 0.606 | 0.5169 - 0.6947 | 0.864 | 0.330 | 0.440 | 0.800 | 0.583 | 0.216 |
|  | ExtraTrees | 0.654 | 0.687 | 0.6046 - 0.7704 | 0.610 | 0.680 | 0.537 | 0.742 | 0.571 | 0.285 |
|  | KNN | 0.628 | 0.615 | 0.5261 - 0.7044 | 0.441 | 0.742 | 0.510 | 0.686 | 0.473 | 0.189 |
|  | SVM | 0.712 | 0.763 | 0.6883 - 0.8385 | 0.627 | 0.763 | 0.617 | 0.771 | 0.622 | 0.389 |
|  | NaiveBayes | 0.660 | 0.681 | 0.5957 - 0.7660 | 0.661 | 0.660 | 0.542 | 0.762 | 0.595 | 0.312 |
|  | GradientBoosting | 0.609 | 0.629 | 0.5414 - 0.7175 | 0.661 | 0.577 | 0.487 | 0.737 | 0.561 | 0.231 |
|  | LightGBM | 0.660 | 0.674 | 0.5862 - 0.7610 | 0.610 | 0.691 | 0.545 | 0.744 | 0.576 | 0.295 |
|  | MLP | 0.660 | 0.720 | 0.6390 - 0.8002 | 0.746 | 0.608 | 0.537 | 0.797 | 0.624 | 0.344 |
|  | XGBoost | 0.609 | 0.674 | 0.5890 - 0.7596 | 0.780 | 0.505 | 0.489 | 0.790 | 0.601 | 0.282 |
|  | AdaBoost | 0.487 | 0.556 | 0.4645 - 0.6477 | 0.915 | 0.227 | 0.419 | 0.815 | 0.574 | 0.182 |

AUC: area under the curve, 95% CI: 95% confidence intervals, PPV: positive predictive values, NPV: negative predictive values, MCC: Matthews Correlation Coefficient.

Supplementary Fig. 1 ROC curves of the ten classifiers in the training and test cohorts of the intra-thrombus model.

Supplementary Fig. 2 ROC curves of the ten classifiers in the training and test cohorts of the peri-thrombus model.

Supplementary Fig. 3 ROC curves of the ten classifiers in the training and test cohorts of the combined model 1.
